# 1 Versus 2-cm Excision Margins for pT2-pT4 Primary Cutaneous Melanoma (MelMarT): A Feasibility Study

**DOI:** 10.1245/s10434-018-6470-1

**Published:** 2018-05-30

**Authors:** Marc D. Moncrieff, David Gyorki, Robyn Saw, Andrew J. Spillane, Howard Peach, Deemesh Oudit, Jenny Geh, Peter Dziewulski, Ewan Wilson, Paolo Matteucci, Rowan Pritchard-Jones, Roger Olofsson Bagge, Frances C. Wright, Nic Crampton, Oliver Cassell, Navid Jallali, Adam Berger, John Kelly, Stephen Hamilton, Amer Durrani, Serigne Lo, Elizabeth Paton, Michael A. Henderson

**Affiliations:** 1 Norfolk & Norwich University Hospital, Norwich, UK; 2 Peter MacCallum Cancer Centre, Melbourne, Australia; 3 Melanoma Institute Australia, Sydney, Australia; 4 Leeds Teaching Hospitals, Leeds, UK; 5 Christie NHS Trust, Manchester, UK; 6 Guy’s & St Thomas’s NHS Trust, London, UK; 7 St Andrew’s Centre for Burns & Plastic Surgery, Chelmsford, UK; 8 North Bristol NHS Trust, Bristol, UK; 9 Hull & East Yorkshire NHS Trust, Hull, UK; 10 Mersey Centre for Burns & Plastic Surgery, Liverpool, UK; 11 Sahlgrenska University Hospital, Göteborg, Sweden; 12 Sunnybrook Health Sciences Centre, Toronto, Canada; 13 Gold Coast Melanoma Clinic, Queensland, Australia; 14 Oxford University Hospitals NHS Trust, Oxford, UK; 15 Imperial Hospital NHS Trust, London, UK; 16 Jefferson University Hospitals, Philadelphia, USA; 17 The Alfred Hospital, Melbourne, Australia; 18 Royal Free Hospital NHS Trust, London, UK; 19 Cambridge University Hospitals, Cambridge, UK; 20 Australia & New Zealand Melanoma Trials Group, North Sydney, Australia

## Abstract

**Background:**

There is a lack of consensus regarding optimal surgical excision margins for primary cutaneous melanoma > 1 mm in Breslow thickness (BT). A narrower surgical margin is expected to be associated with lower morbidity, improved quality of life (QoL), and reduced cost. We report the results of a pilot international study (MelMarT) comparing a 1 versus 2-cm surgical margin for patients with primary melanoma > 1 mm in BT.

**Methods:**

This phase III, multicentre trial [NCT02385214] administered by the Australia & New Zealand Medical Trials Group (ANZMTG 03.12) randomised patients with a primary cutaneous melanoma > 1 mm in BT to a 1 versus 2-cm wide excision margin to be performed with sentinel lymph node biopsy. Surgical closure technique was at the discretion of the treating surgeon. Patients’ QoL was measured (FACT-M questionnaire) at baseline, 3, 6, and 12 months after randomisation.

**Results:**

Between January 2015 and June 2016, 400 patients were randomised from 17 centres in 5 countries. A total of 377 patients were available for analysis. Primary melanomas were located on the trunk (56.9%), extremities (35.6%), and head and neck (7.4%). More patients in the 2-cm margin group required reconstruction (34.9 vs. 13.6%; *p* < 0.0001). There was an increased wound necrosis rate in the 2-cm arm (0.5 vs. 3.6%; *p* = 0.036). After 12 months’ follow-up, no differences were noted in QoL between groups.

**Discussion:**

This pilot study demonstrates the feasibility of a large international RCT to provide a definitive answer to the optimal excision margin for patients with intermediate- to high-risk primary cutaneous melanoma.

Following a diagnosis of primary cutaneous melanoma, a secondary wider excision around the original biopsy scar is advocated to reduce risk of local recurrence and improve patient outcomes. Surprisingly, the extent of this elective wide excision is still to be resolved. Guidelines for surgical margins of resection vary internationally, from 1 to 3 cm, depending on Breslow thickness of the primary, which translates into excision defects from 2 to 6 cm in diameter.^1^–^3^ The recommended margins of excision for patients with intermediate- and high-risk primaries is particularly variable, with differing interpretations of the data from two, similarly designed, randomised, controlled trials (RCTs) fuelling the debate.^4^–^8^ The authors of one trial concluded that a narrow, 1-cm margin resulted in increased locoregional recurrence rate translating into a worse disease-specific survival and another group concluded that there was no difference in either locoregional or disease-specific survival with a narrow, 2-cm margin.^4^,^5^ There is a growing concern internationally amongst surgeons that the excess morbidity caused by larger excision defects, including increased hospital stay, complications, and need for reconstructive surgery, may not be necessary, particularly because previous RCTs have shown that local recurrence rates are low, ranging from 1.3% for intermediate-risk primaries to 3.3–4.3% for high-risk primaries.^5^,^9^–^11^

With optimal therapy, approximately 90% of melanoma patients survive beyond 10 years. Because the overwhelming majority of melanoma patients have surgery and no other treatment, quality of life after surgery is a key survivorship issue. More than 110,000 patients are currently alive following a diagnosis of melanoma in the United Kingdom.^12^ Long-term follow-up data of previous RCTs have shown a significant worsening in quality-of-life associated with postoperative morbidity and poor cosmesis from surgical scars.^9^,^13^ A recent, multicentre, retrospective analysis demonstrated that the prevalence of chronic, moderate-severe neuropathic pain was 8% following wide excision for melanoma.^14^

Currently, approximately 40% of all melanoma patients with intermediate- to high-risk primaries are subject to 2- to 3-cm excision margins. However, given the available data, it is reasonable to suspect that a 1-cm margin may be sufficient to achieve local control for over 95% of these patients.^5^,^9^,^11^ The authors of the latest Cochrane review concluded that an appropriately designed trial of an adequate sample size is clearly needed to unify international guidance and to benefit the large and increasing numbers of melanoma patients worldwide.^10^ The purpose of the full study will be to determine whether there is a difference in local recurrence rates and melanoma survival rates for patients treated with either a 1-cm excision margin or 2-cm margin for both intermediate- and high-risk melanomas, with survival outcomes, quality of life, and health economics data as secondary measures. In this paper, we present the feasibility data of the internal pilot study.

## Methods

MelMarT is a registered, phase III, surgical RCT [clinicaltrials.gov registration: NCT02385214] with international ethical/IRB approval [Australian ethical registration number: HREC/14/RPAH/330] administered by the Australia & New Zealand Medical Trials Group (ANZMTG 03.12). Following diagnosis (by shave or excision biopsy) of a primary cutaneous melanoma of Breslow thickness > 1 mm (pT2a-pT4b/AJCC IB-IIC; AJCC 8th edition),^15^ eligible patients were randomised electronically in a 1:1 fashion to either a 1 or a 2-cm wider excision margin. In each arm, patients were staged at the same operation with sentinel lymph node biopsy (SLNB). Patients were stratified according to age, sex, and AJCC stage (intermediate risk: IB-IIA and high risk: IIB-IIC). Review of the primary melanoma histology slides was performed internally at participating institutions by designated dermatopathologists. At the time of definitive surgery, the designated margin was measured from the scar, marked, and photographed for quality assurance. The skin incision was continued vertically down through subcutaneous tissue to the deep fascia, which could be removed en bloc at the surgeon’s discretion. Patients underwent direct primary closure or reconstructive surgery with a local flap or a skin graft according to the preference of the treating surgeon. Patients with positive SLNB were managed according to the treating unit’s local protocol.

Patients’ quality of life was measured using the validated FACT-M questionnaire version 4 at baseline then 3, 6, and 12 months postrandomisation.^16^ Neuropathic pain was measured at the same time points using the validated PainDetect questionnaire.^17^ Health economics data (not reported in this paper) were collected in prespecified centres using EQ 5-D questionnaire with patient-specific financial questionnaires and health resource usage data.^18^

## Results

**Fig. 1:**
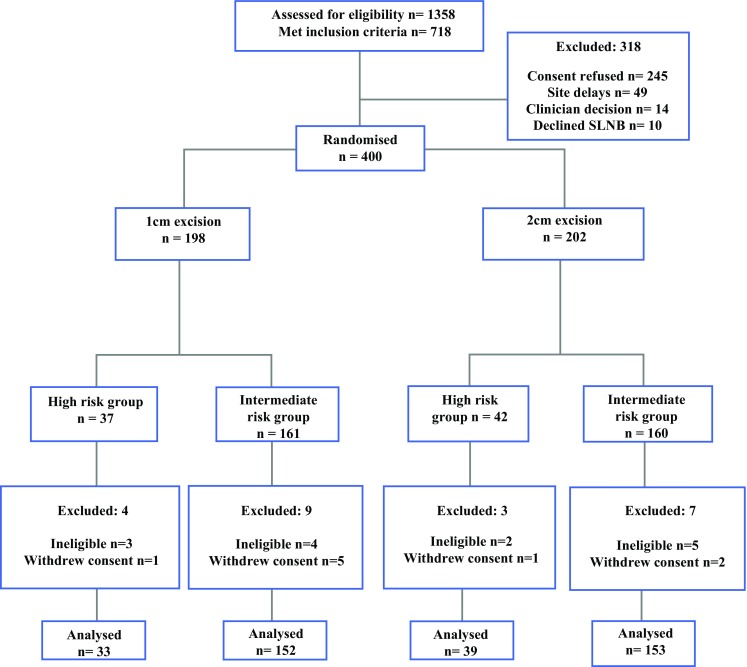
CONSORT diagram for MelMarT

Between January 2015 and June 2016, 400 patients were randomised in 17 centres across 5 countries. The database was locked and analysed according to the predesignated statistical plan once the last patient randomised had completed 12 months follow-up and completed their quality of life data (June 2017). Figure 1 shows the CONSORT diagram. Comprehensive screening data were available from the majority of the recruiting centres (Fig. 1). In total, 1358 patients were screened of which 718 (52.9%) met the inclusion criteria. Of these, 318 were not enrolled; 245 (77.0%) patients declined to be enrolled, 49 (15.4%) patients were unable to undergo the treatment intervention within the protocol-prescribed timeframe, 14 (4.4%) patients were not deemed suitable for the trial by the clinician, and 10 (3.1%) patients declined the sentinel lymph node biopsy procedure.

In this study, 377 patient datasets were available for analysis (23 patients were deemed ineligible or withdrew consent). Table 1 indicates the details of the patient demographics and tumour characteristics. Both cohorts were well-matched with no significant differences. The majority of the lesions were located on the torso (56.9%), followed by the extremities (35.6%) and the head and neck region (7.4%). The rate of positive SLNB was 15.2% in the 1-cm group and 22.9% in the 2-cm group (absolute difference: 7.7%; *p* = 0.058).

**Table 1:** Patient and tumour characteristics

Patient characteristics	1 cm (*n* = 185)	2 cm (*n* = 192)	Total (*n* = 377)
*Gender*			
Male	104 (56.2%)	107 (55.7%)	211 (54.5%)
Female	81 (43.8%)	85 (44.3%)	186 (45.5%)
*Age (year)*			
Mean (SD)	58.97 (± 13.10)	58.19 (± 13.21)	58.50 (± 13.15)
*Age (year)*			
< 45	28 (15.1%)	28 (14.6%)	56 (14.5%)
45–65	86 (46.5%)	89 (46.4%)	175 (45.2%)
> 65	71 (38.4%)	75 (39.1%)	146 (40.3%)
*BMI*			
Mean (SD)	28.43 (± 6.56)	28.38 (± 5.20)	28.40 (± 5.88)
*ECOG score*			
0	173/181 (95.6%)	176/187 (94.1%)	349 (94.8%)
1	8/181 (4.4%)	11/187 (5.9%)	19 (5.2%)

Tumour characteristics	1 cm (*n* = 185)	2 cm (*n* = 192)	Total (*n* = 377)
*Breslow thickness (mm)*			
Mean (SD)	2.12 (± 1.17)	2.27 (± 1.39)	2.20 (± 1.28)
Min max	1.0, 7.5	1.0, 8.5	1.0, 8.5
*Breslow thickness (mm)*			
1.0–2	111 (60.0%)	112 (58.3%)	223 (59.2%)
2.1–4	61 (33.0%)	60 (31.3%)	121 (32.1%)
> 4	13 (7.0%)	20 (10.4%)	33 (8.7%)
*Mitotic rate*			
Mean (SD)	4.81 (± 5.26)	4.88 (± 5.07)	4.84 (± 5.16)
*Ulceration*			
Present	47 (25.4%)	52 (27.1%)	99 (26.3%)
Absent	138 (74.6%)	138 (71.9%)	276 (73.2%)
Unknown	0 (0.0%)	2 (1.0%)	2 (0.5%)
*Location*			
Head and neck	12 (6.5%)	16 (8.9%)	28 (7.4%)
Axial	102 (55.4%)	112 (58.3%)	214 (56.9%)
Extremity	70 (38.0%)	64 (33.3%)	134 (35.6%)
*Sentinel node status*			
Positive	28 (15.2%)	44 (22.9%)	72 (19.1%)
Negative	156 (84.8%)	148 (77.1%)	304 (89.9%)

Table 2 indicates the reconstructive burden across the two cohorts; 34.9% patients required reconstruction with a skin graft or local flap in the 2-cm group compared with 13.6% in the 1-cm group [*p* < 0.0001; odds ratio (OR) 3.4 (2.0–5.8)]. There was a significantly increased need for reconstruction in the 2-cm group at all locations, especially the extremities and head and neck.

**Table 2:** Reconstruction rates by cohort and anatomical location

Reconstruction? (Y/N)	1 cm (*n* = 184)	2 cm (*n* = 192)	Total (*n* = 376)	Significance
*Any site*				
Yes	25 (13.6%)	67 (34.9%)	92 (24.5%)	*p* < 0.0001 OR 3.4 [2.0–5.8]
No	159 (86.4%)	125 (65.1%)	284 (75.5%)	
*By primary location*				
*Head and neck*				
Yes	1 (8.3%)	11 (68.8%)	12 (42.9%)	*p* = 0.002 OR 19.3 [2.6–566.3]
No	11 (92.6%)	5 (31.2%)	16 (57.1%)	
*Axial*				
Yes	15 (14.7%)	29 (25.9%)	44 (20.6%)	*p* = 0.043 OR 2.0 [1.0–4.1]
No	87 (85.3%)	83 (74.1%)	170 (79.4%)	
*Extremity*				
Yes	9 (12.9%)	27 (42.2%)	36 (26.9%)	*p* = 0.0003 OR 4.8 [2.1–12.1]
No	61 (87.1%)	37 (57.8%)	98 (73.1%)	

**Fig. 2:**
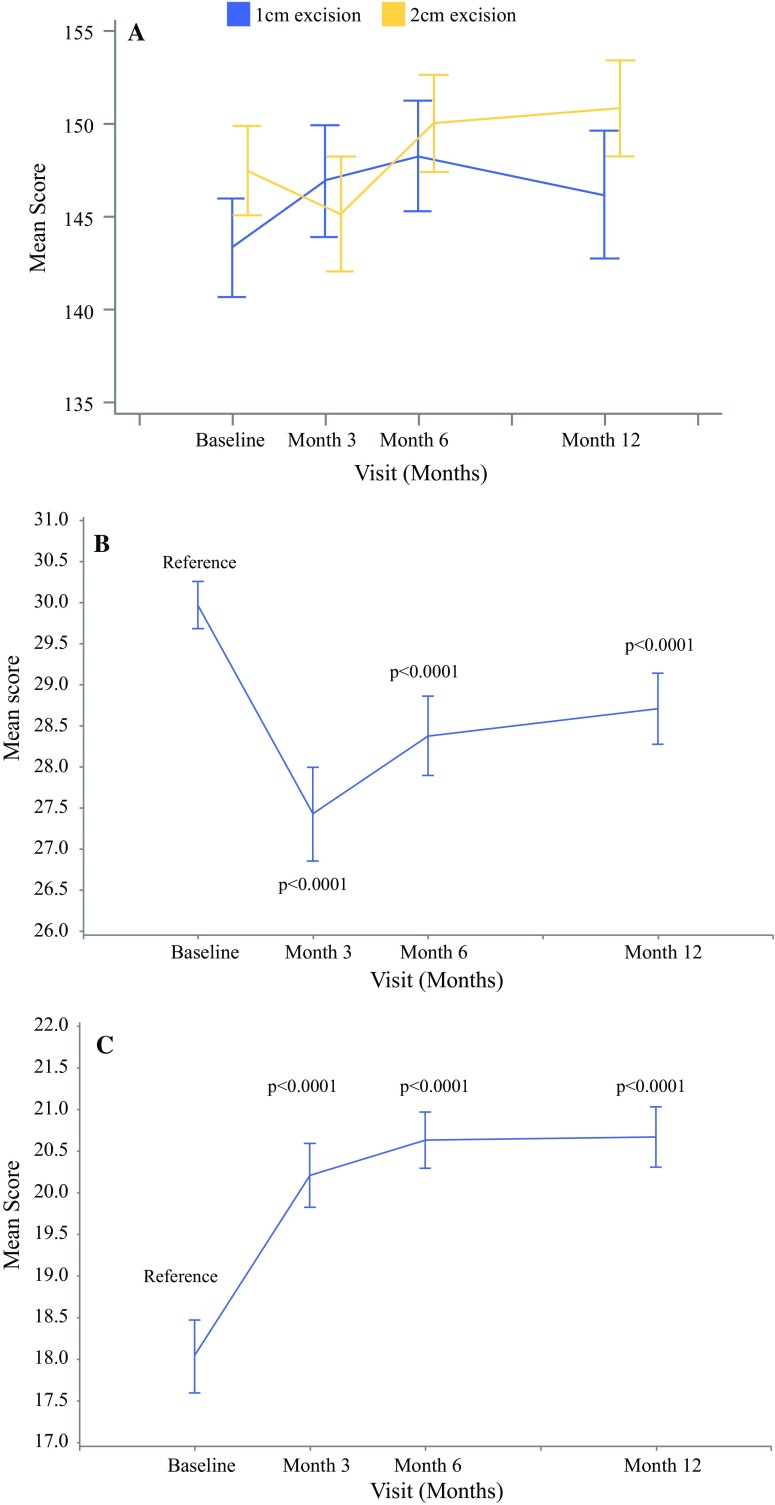
**a** Mean FACT-M total scores (1 vs. 2-cm excision margins)**. b** FACT-M melanoma surgery subscale scores (all patients). **c** FACT-M emotional well-being scores (all patients)

The quality of life data indicated no difference from baseline at any time point for the majority of the FACT-M subscales neither within nor between the randomisation groups, nor on subgroup analysis. The exceptions were the “melanoma surgery” subscale, which showed a significant and sustained decrease in score (indicating a worse quality of life for this subscale) from baseline (*p* < 0.0001 at 3, 6, and 12 months with reference to baseline) and the “emotional well-being” subscale, which showed a significant and sustained increase in score (indicating an improved quality of life for this subscale) from baseline (*p* < 0.0001 at 3, 6, and 12 months with reference to baseline; Fig. 2a–c). There was no difference between the 1 and 2-cm arms in these two subscales. Neuropathic pain score analysis indicated a significant but transient increase in pain level; the strongest pain and highest average pain scores were recorded at 3 months compared with baseline across both 1 and 2-cm groups. The scores returned to baseline at 6 and 12 months.

Table 3 outlines the perioperative surgical adverse events data at the wide excision sites. The overall treatment-related surgical adverse event rate was 10.3% in the 1-cm arm and 11.4% in the 2-cm arm (difference not significant). There was a significant increase in wound necrosis in the 2-cm arm compared with the 1-cm arm (3.6 vs. 0.5%, *p* = 0.036). With one exception (a haematoma in the 2-cm arm; grade IIIa), the adverse events were minor or mild: grade I–II in both the 1 and 2-cm arms. In follow-up, the melanoma-related surgical adverse event rate was 5.4% in the 1-cm arm and 3.6% in the 2-cm arm (difference not significant).

**Table 3:** Surgical adverse events at wide excision site

Surgical adverse event (Clavien-Dindo grade)	1 cm *n* (events)	%	2 cm *n* (events)	%
*Wound dehiscence*	4	2.2	4	3.2
*Haematoma*	3	1.6	2	1
Grade I	3	1.6	1	0.5
Grade IIIa	0		1	0.5
*Haemorrhage*	0		0	
*Wound infection*	11	5.9	9	4.7
Grade I	1	0.5	3	1.6
Grade II	10	5.4	6	3.1
*Wound necrosis (including partial/total loss of skin graft)*	1	0.5	7	3.6*
Grade I	0		6	3.1
Grade II	1	0.5	1	0.5
*Total*	19	10.3	22	11.4

*p = 0.036

## Discussion

In this study, we have presented the feasibility data and initial quality of life outcomes data for the internal pilot study for MelMarT, a large phase III RCT that requires a sample size of nearly 10,000 patients to test the coprimary endpoints of local recurrence and melanoma-specific survival. A noninferiority statistical design is required to prove parity in terms of safety and efficacy of the clinical endpoints between the 1 and 2-cm arms, which, combined with the relatively low event rate of the primary outcome of local recurrence, inflates the sample size greatly compared with a superiority design.^19^ Accordingly, before embarking on this large endeavour, it was necessary to conduct an internal pilot to test the robustness of the protocol and recruitment rates across multiple centres internationally.

Unlike previous RCTs performed to assess margins, the MelMarT study mandates SLNB as eligibility criteria.^5^,^9^,^11^,^20^–^22^ This ensures optimal staging and risk stratification. It was interesting to note that, despite careful stratification according to patient and primary tumour characteristics, the absolute difference in SLNB positivity between the two cohorts was 7.7%, which was a near-significant finding (*p* = 0.058). These data highlight and lend weight to the concerns that have been raised regarding the interpretation of the results of previous RCTs.^4^,^7^,^11^ In particular, Hayes et al. proposed that the findings in their long-term analysis, demonstrating a worse clinical outcome, were linked directly to their previous finding of increased locoregional recurrence associated with a narrower 1-cm excision margin compared with a 3-cm excision margin.^4^,^11^ However, in both surgical groups, the incidence of nodal recurrence outweighed the incidence of local recurrence by at least 5–1. An alternative explanation, that the excess nodal disease in the narrow margin group was indicative of poor prognostic disease before the intervention, rather than resulting from the narrow margin intervention itself, has been suggested.^7^,^23^

### Clinical Outcomes

The trial management committee deemed that it was not appropriate to present any outcomes data related to the primary endpoints after 1 year of follow-up. The major clinical finding was that there was a significant increase in the use of reconstructive procedures between the two cohorts (Table 2). Overall, the rate of reconstruction was more than doubled in the 2-cm arm compared with the 1-cm arm (39.4 vs. 13.6%, respectively; *p* < 0.0001). The largest difference was seen in the head and neck region (1 cm: 8.3% vs. 2 cm: 68.8%; *p* = 0.002), although the subgroup sample size was small and the confidence intervals (CI) were wide; thus, the size of the difference needs to be interpreted with caution. Similarly, there was a large and significant difference in the incidence of reconstruction in the extremities. Clinically, these data are relevant, given the relative lack of tissue laxity in the extremities, the cosmetic implications of large excision margins in the head and neck region and the unique functional and anatomical considerations of both areas. It is interesting to note that only one prior RCT included patients with head and neck cutaneous melanoma, comprising only 0.3% (16/326) of cases in that particular study and < 0.02% of all participants in the pooled RCT evidence to date.^22^ Two of the previous RCTs reported a significantly increased need for reconstruction when comparing a 2-cm wider excision margin with a 4-cm margin. In the Scandinavian study, it was possible to close the wound directly in 69% of the 2-cm group, which is close to our findings (65.1%).^5^ In the Intergroup and the MSLT-1 studies, the overall reconstruction rates were 28 and 22% respectively, again similar to our own data.^9^,^24^ Haigh et al. performed a systematic review of all the available data at the time and estimated that the number needed to harm from a wider excision was 3 (95% CIs 2.38–3.7), indicating that for every three patients undergoing a wider excision, one patient would undergo a reconstruction who would otherwise not require it if a narrower margin had been used.^25^ In our dataset, the number needed to harm was calculated as 4.69 (95% CIs 3.45–8.1), indicating a substantial reconstructive burden that could be avoided with the use of a narrower 1-cm margin compared to a 2-cm margin.

### Quality of Life Outcomes

Our QOL data yielded interesting and possibly surprising results. Ultimately there was no difference in quality of life or neuropathic pain data in any domain between the 1 and 2-cm groups. Similarly, there were no differences between the two margins in any subgroup analyses. One RCT QOL analysis was published from the UK BAPS/MSG study comparing 1 versus 3-cm margins for thicker melanoma.^13^ The wider margin was associated with a worse QOL initially, which normalised to baseline after 6 months. This was the case for both the mental and physical component scores of QOL tool employed. In our study, we noticed a significant worsening of the FACT-M melanoma surgery subscale, which persisted after 12 months (Fig. 2). Similarly, the emotional well-being subscale progressively improved over the 12-month postoperative period. The differences between our study and the U.K. BAPS/MSG study may be due to the different QOL tools used, although the improvement in emotional well-being may be representative of the improved multidisciplinary care and support most patients currently receive in major cancer centres.

### Adverse Events

We found that the surgical adverse event rate (AER) was nearly identical for both arms of the study: approximately 10–11%. The complication rate was the same between the two arms of the study except the wound necrosis/skin graft loss rate. We suggest that this is related to the increased rate of reconstruction in the 2-cm arm. The wide excision biopsy site surgical AER was 5.4 and 3.6% in the 1 and 2-cm groups, respectively. In comparison, the Intergroup trial demonstrated an approximate 5% complication rate at the primary site, regardless of margin.^9^ The Sunbelt Melanoma Trial demonstrated a 4.6% surgical AER at the sentinel node biopsy site, which is comparable to our data.^26^ Nearly all surgical AEs recorded in our study were grade I or II indicating that the procedures were performed to a uniformly high standard in the recruiting centres.

### Recruitment

Our data indicate that the pilot study had a high rate of recruitment with the majority of potentially eligible patients declining participation rather than not being offered the trial. These are encouraging data and are likely to be due to both the permissive trial design, allowing patients to be recruited to subsequent trials upon progression or discovery of a positive sentinel node biopsy, and the relative lack of competing clinical trials for patients who are at the same stage of the disease. Furthermore, the successful completion of the pilot study indicates enthusiastic engagement by clinicians and consumer groups internationally who are keen to see the issue resolved for the benefit of future patients. A simple trial design with the experimental intervention representing a seemingly modest modification of the internationally accepted standard of care also lends itself greatly to successful recruitment.

## Conclusions

We have shown that the MelMarT study design is feasible and straightforward to recruit to and implement. Prospective, future patients and clinicians would benefit from this information in the preoperative consultation to aid undertaking informed consent. The rate of reconstruction is significantly increased when a wider margin is employed, and this is consistent with previous RCTs. This information that can be used immediately in clinical decision-making, particularly where local recurrence rates are very low, namely the pT2 subgroup of patients. In summary, the internal pilot of MelMarT has been successful, indicating that the international phase III trial should proceed with only minor amendments to the protocol.
